# Reciprocal Relationships Between Problematic Social Media Use, Problematic Gaming, and Psychological Distress Among University Students: A 9-Month Longitudinal Study

**DOI:** 10.3389/fpubh.2022.858482

**Published:** 2022-04-08

**Authors:** Ching-Wen Chang, Ru-Yi Huang, Carol Strong, Yi-Ching Lin, Meng-Che Tsai, I-Hua Chen, Chung-Ying Lin, Amir H. Pakpour, Mark D. Griffiths

**Affiliations:** ^1^Graduate Institute of Social Work, National Taiwan Normal University, Taipei, Taiwan; ^2^Department of Family and Community Medicine, E-Da Hospital, Kaohsiung, Taiwan; ^3^School of Medicine for International Students, College of Medicine, I-Shou University, Kaohsiung, Taiwan; ^4^Department of Public Health, College of Medicine, National Cheng Kung University, Tainan, Taiwan; ^5^Department of Early Childhood and Family Education, National Taipei University of Education, Taipei, Taiwan; ^6^Department of Pediatrics, College of Medicine, National Cheng Kung University Hospital, National Cheng Kung University, Tainan, Taiwan; ^7^Department of Medical Humanities and Social Medicine, College of Medicine, National Cheng Kung University, Tainan, Taiwan; ^8^Chinese Academy of Education Big Data, Qufu Normal University, Qufu, China; ^9^College of Medicine, Institute of Allied Health Sciences, National Cheng Kung University Hospital, National Cheng Kung University, Tainan, Taiwan; ^10^Department of Occupational Therapy, College of Medicine, National Cheng Kung University, Tainan, Taiwan; ^11^Biostatistics Consulting Center, College of Medicine, National Cheng Kung University Hospital, National Cheng Kung University, Tainan, Taiwan; ^12^Department of Public Health, College of Medicine, National Cheng Kung University, Tainan, Taiwan; ^13^Department of Nursing, School of Health and Welfare, Jönköping University, Jönköping, Sweden; ^14^International Gaming Research Unit, Psychology Department, Nottingham Trent University, Nottingham, United Kingdom

**Keywords:** problematic social media use, anxiety, depression, problematic gaming, longitudinal study

## Abstract

**Background:**

The causal relationships between two specific types of problematic use in internet-related activities [i.e., problematic social media use (PSMU) and problematic gaming (PG)] and psychological distress remain controversial. The present study investigated the temporal relationships between PSMU, PG, and psychological distress (i.e., anxiety, depression) in university students.

**Methods:**

Hong Kong and Taiwan university students [*N* = 645; n_male_ = 266; mean = 20.95 years (SD = 5.63)] were recruited for a survey study, with follow-ups at 3, 6, and 9 months after baseline assessment. The Bergen Social Media Addiction Scale, Internet Gaming Disorder Scale-Short Form, and the Hospital Anxiety and Depression Scale were used to assess studied variables. Demographics including age, physical characteristics (i.e., height, weight, and body mass index), and cigarette use were compared between participants who completed all the follow-ups and those who dropped out. Random intercept cross-lagged models were constructed to understand the reciprocal relationships between PSMU, PG, and psychological distress.

**Results:**

No significant differences were found in age, physical characteristics, and cigarette use between participants who completed all the follow-ups and those who dropped out. Findings indicated that a high level of PSMU significantly increased the level of anxiety and a high level of anxiety significantly increased the level of PSMU. A high level of PSMU significantly increased the level of depression but the level of depression did not significantly affect the level of PSMU. A high level of PG significantly increased the level of anxiety, but the level of anxiety did not significantly affect the level of PG. A high level of depression significantly increased the level of PG, but the level of depression did not significantly affect the level of PG.

**Conclusion:**

The patterns of the causal relationship between PIU and psychological distress variables differ. A reciprocal relationship was only found between the level of PSMU and the level of anxiety. Moreover, the longitudinal design found no differences in the waves in terms of gaming by the participants.

## Introduction

With the increasing connectivity offered by digital devices and online platforms, there is a growing concern about the effect of problematic internet use (PIU). A recent literature review indicated that the worldwide prevalence of internet addiction among college students ranged from 4% to 25% (1). A meta-analysis examining the prevalence of PIU in 31 nations across seven world regions for all populations estimated an overall prevalence of 6.0% (2). Moreover, individuals who excessively use the internet are more likely to have mental health issues (3–6).

Although prior research indicates that PIU can be classed into generalized PIU (i.e., total problematic online behavior that may encompass many online activities rather than one behavior specifically) and specific PIU (i.e., problems with specific online applications, such as social media use, gaming, and gambling) (7–9), there is a growing consensus that individuals tend to have preferred online activities and that the patterns of different types of online activities for an individual are not comparable (10–18). Therefore, the present study does not use PIU to represent any specific type of problematic use in internet-related activities. Instead, the present study directly focuses on problematic social media use (PSMU) and problematic gaming (PG). Both social media and internet games are easily accessed and can provide psychological rewards to the users. PSMU and PG may share specific characteristics including compulsive use, mood modification, and the continuation despite negative consequences (19). However, social media use may constitute a more ongoing process than online gaming because online gaming may have more well-defined beginnings and endings for each game. Moreover, while online gaming contains similar stimuli and one of the major goals for online gaming is to win a game, the aims of social media use involve social interactions, which demands more emotional effort (18). Studies have shown that PSMU is associated with depression and anxiety [e.g., (20–24)]. PG has also been found to be associated with depression, anxiety, attention deficit/hyperactivity, loneliness, and social phobia (4, 20, 25, 26).

There are two commonly cited conceptual frameworks to explain the causal relationship between PIU and psychological distress. The first framework posits that experiencing psychological distress could lead to PIU (27–29). Mores specifically Davis's (30) cognitive behavioral model proposes that psychological distress, such as depression and anxiety, is an essential and significant factor in the development of problematic internet use. Internet use can be a coping strategy for psychological distress. In order to be distracted from their problems and negative emotions, internet users with high psychological distress can become overly involved in online activities, such as online gaming (31). For those with psychological distress due to issues related to face-to-face social interactions, social media use allows users to connect with others and provide them with sense of autonomy in social interactions (32, 33). Once internet users find online activities psychological rewarding, their expectation of receiving such type of reward can drive them to continuous internet use (32, 34).

In terms of the second framework, several researchers have noted that PIU could contribute to psychological distress (35–37). Excessive internet use can lead to disruptions in personal development in real life because over-involvement in online activities, including online gaming and social media use, and can cause a decrease in time that is spent on career or academic development (38, 39). Preoccupation with online activities can also lead to social withdrawal from the real world and impair offline social relationships (39–42). Such disruptions and social impairments, resulting from PIU, can have negative impacts on psychological wellbeing (37, 43, 44).

Although the association between PIU and psychological distress has been well-established in many studies, little is known regarding their causality (45) due to a great majority of prior studies utilizing cross-sectional designs [e.g., (46, 47)]. In recent years, a few studies have attempted using longitudinal designs to advance knowledge in this field. For example, among longitudinal studies investigating relationship between social media use and psychological distress among adolescents, the majority only examined a one-way directional effect (i.e., from social media use to mental health) among adolescents. Results have indicated that more time spent using social media might lead to depression (48–50). However, these studies lack measures for subjective experience of social media use. Recently, with an attempt to investigate whether the relationship was bi-directional, Li et al. (51) collected data at two time points and found that having depression was associated with later PSMU, and that PSMU was also related to later development of depression in adolescents. However, due to the limitation of two time points of data collection, the study findings did not provide sufficient evidence to support causality and reflect a reciprocal relationship between PSMU and depression over time.

A few longitudinal studies examined causal relationship between PG and psychological distress and found that PG predicts later psychological distress, including depression and anxiety (52–54). Teng et al. (55), investigated causal relationship during COVID-19 pandemic and found that depression and anxiety predicted internet gaming disorder and video game use. These studies also had the limitation of two time points of data collection. A recent Norwegian study used three waves, of data from adolescents to investigate causality between pathological gaming and psychological distress (56). Findings indicated that there was a reciprocal relationship between pathological gaming and depression. In addition, the study results also showed that pathological gaming led to anxiety.

Although findings of aforementioned longitudinal studies provide some information on the temporal relationship between PIU and psychological distress, there are two major limitations for these studies. First, the causal relationship between anxiety (a type of psychological distress) and PSMU has been understudied. Second, a majority of longitudinal studies have focused on adolescent samples. Studies investigating the causality between PSMU, PG, and psychological distress among university students are lacking.

To address these knowledge gaps, the present study investigated temporal relationship between two of the most common types of psychological distress (i.e., anxiety, depression) and two specific types of problematic use in internet-related activities (i.e., PSMU, PG) among university students, using a longitudinal study design with data collection at three time-points. Moreover, given that longitudinal data usually have the issues of loss to follow-up, the present study also compared the demographic information [including age, body mass index (BMI), and cigarette use] between retained and drop-out samples. A clear picture concerning the causality between PSMU, PG, and psychological distress not only could advance the knowledge on contributors and consequences of PIU, but could also provide insights for policymakers, university managers, and clinicians to develop effective interventions to address issues pertaining to internet use among university students. If the relationships between PIU and psychological distress are reciprocal, intervention and prevention programs aimed to address PIU should pay attention to breaking the vicious cycle between PIU and psychological distress. On the other hand, if the relationship between PIU and psychological distress is unidirectional, identifying which one is the antecedent would provide additional insight on the focus of prevention/intervention for addressing the outcome variable.

## Methods

### Participants and Procedure

After obtaining approval from the ethics committee of a Hong Kong university (Ref. No. HSEARS20171212001), a *Google Forms* online survey was generated by the authors and the survey began with details of the study's purpose and requirements. The second page of the survey asked whether the participants were willing to participate and asked for their informed consent. The survey was shut down immediately if the participants did not provide informed consent.

The target participants were university students studying in Hong Kong or Taiwan and they received the online survey information through the dissemination from several research assistants and teaching faculties. More specifically, the research assistants and teaching faculties disseminated the hyperlink or the QR code that led to the first page of the survey to potential participants. For Hong Kong university students, the faculties included health and social sciences, humanities, engineering, design, applied science and textiles, business, construction and environment, and hotel and tourism management. For Taiwan university students, the faculties included liberal arts, science, management, engineering, electrical engineering and computer science, social science, bioscience and biotechnology, and medicine. The potential participants were those who attended university lectures comprising liberal education.

The baseline assessment was conducted between March and June 2018. Eligible participants were those aged 18 years or above, being capable of understanding written Chinese in traditional characters, and possessing a smartphone with internet access. The survey requested each participant to provide their smartphone number together with a frequently used email account to contact them for the three follow-up participations every 3 months (i.e., at 3-month, 6-month, and 9-month follow-up). Smartphone number and email accounts assisted the present authors in excluding individuals who completed the survey twice or more at each assessment time point. Incentives were given to those who completed the survey two to four times. Hong Kong university students received 350 Hong Kong Dollars (HKD; 7.8 HKD ≈ 1 USD) if they completed the survey four times, 150 HKD if they completed the survey three times, and 50 HKD if they completed the survey twice. Taiwan university students received 350 New Taiwan Dollars (NTD; 30 NTD ≈ 1 USD) if they completed the survey four times, 150 NTD if they completed the survey three times, and 50 NTD if they completed the survey twice. No financial incentives were provided for those who only completed the survey once.

At baseline assessment, 800 eligible students were approached and 645 completed the baseline survey (309 from Hong Kong and 336 from Taiwan; response rate: 80.6%). A total of 178 students (27.6%) completed surveys at all four assessment time points; 94 completed surveys at three assessment time points (14.6%); 86 completed surveys at two assessment time points (13.3%); and 287 only completed the survey at baseline assessment (44.5%).

### Measures

#### Bergen Social Media Addiction Scale

The six-item BSMAS assessed the level of PSMU (20). The six items corresponding to six core addiction components (i.e., salience, mood modification, tolerance, withdrawal, conflict, and relapse) proposed by Griffiths (57). Each BSMAS item evaluated how an individual experienced the use of social media during past month. A BSMAS sample item is “You feel an urge to use social media more and more” with the response options being “very rarely, rarely, sometimes, often, and very often.” All the items were rated on a five-point Likert scale with total scores ranging between 6 and 30. A higher BSMAS total score indicated a higher level of PSMU (58). Satisfactory psychometric properties of the BSMAS such as high internal consistency and confirmed unidimensional structure have been found in previous studies [e.g., (21, 59–61)]. Furthermore, the measurement invariance of the BSMAS has been supported across Hong Kong and Taiwan university students (62). The Cronbach's α of the BSMAS in the present study was 0.81 (baseline), 0.84 (first follow-up), 0.84 (second follow-up), and 0.84 (third follow-up).

#### Internet Gaming Disorder Scale-Short Form

The nine-item IGDS-SF9 assessed PG (63, 64) with each item corresponding to the nine IGD criteria described in the DSM-5 (65). Each IGDS-SF9 item evaluated how an individual experienced gaming during the past month. An IGDS-SF9 sample item is “Do you feel preoccupied with your gaming behavior?” with the response options being “never, rarely, sometimes, often, and very often.” All the items were rated on a five-point Likert scale with total scores ranging between 9 and 45. A higher IGDS-SF9 total score indicated a higher level of PG (66). Satisfactory psychometric properties of the IGDS-SF9 such as high internal consistency and confirmed unidimensional structure have been found in previous studies [e.g., (21, 61, 66–73)]. Furthermore, the measurement invariance of the IGDS-SF9 has been supported among Hong Kong and Taiwan university students (62). The Cronbach's α of the IGDS-SF9 in the present study was 0.92 (baseline), 0.91 (first follow-up), 0.91 (second follow-up), and 0.92 (third follow-up).

#### Hospital Anxiety and Depression Scale

The 14-item HADS assessed two types of psychological distress (i.e., anxiety and depression) (74). Seven HADS items assessed anxiety and seven HADS items assessed depression during the past 2 weeks. A HADS anxiety sample item is “I feel tense or wound up” with the response options being “most of the time, a lot of the time, from time to time occasionally, and not at all.” A HADS depression sample item is “I feel as if I am slowed down” with the response options being “nearly all the time, very often, sometimes, and not at all.” All 14 items were rated on a four-point Likert scale with scores ranging between 0 and 21 for each type of psychological distress. A higher score on each subscale indicated a higher level of anxiety and/or depression, respectively (74). Satisfactory psychometric properties of the HADS such as high internal consistency and confirmed unidimensional structure have been found in previous studies [e.g., (74, 75)]. Furthermore, the measurement invariance of the HADS has been supported among Hong Kong and Taiwan university students (76). The Cronbach's α of the HADS anxiety subscale in the present study was 0.80 (baseline), 0.81 (first follow-up), 0.79 (second follow-up), and 0.81 (third follow-up); HADS depression subscale was 0.65 (baseline), 0.70 (first follow-up), 0.71 (second follow-up), and 0.79 (third follow-up).

### Data Analysis

Descriptive statistics were used to analyze socio-demographic and internet use characteristics of the participants. Weight, height, and BMI were assessed to investigate whether the present sample contained a higher BMI that may contribute to psychological distress among individuals. Attrition analysis was conducted to examine whether significant differences in demographics existed between participants who completed all the follow-ups and those who dropped out. More specifically, age, BMI, and cigarette use were compared between the two groups. Age, BMI, and cigarette use were compared because they can be important confounding factors in relation to individuals' psychological distress (77–81). Pearson correlations were used to understand the bivariate association between studied variables. Structural equation modeling (SEM) was utilized to examine the impacts of PSMU and PG, respectively, on distress (anxiety or depression) or vice versa. Before conducting the Pearson correlations and SEM, imputation was conducted to take into account the missing data. The imputation was acceptable because the analytic approaches that exclude participants with missing data may lead to estimation biases in statistical results and loss of precision (82, 83).

For the data imputation, the Monte Carlo Markov chain (MCMC) method in the PRELIS program of the LISREL was used to conduct multiple imputations. The multiple imputations used the variables of PSMU, PG, depression, and anxiety in each study wave to estimate the imputed data. The dataset with missing data imputed was then transformed into a polychoric correlation matrix (PCM) and asymptotic covariance matrix (ACM) for use in LISREL. With these two matrices, the estimation of diagonally weighted least square was adopted in LISREL to perform random intercept cross-lagged model (please see detailed information of the random intercept cross-lagged model below).

In the random intercept cross-lagged models using longitudinal data (84, 85), the following associations were correlated: (1) relationship between the same variable across four time points (e.g., anxiety affected only anxiety; PSMU affects only PSMU); (2) relationship between the different measures from the same wave (e.g., anxiety at Time 1 correlated with PSMU at Time 1); and (3) lagged effects from the proposed cause at each time point to its subsequent proposed result at each time point (e.g., anxiety at Time 1 additionally affected PSMU at Time 2; PSMU at Time 1 additionally affected anxiety at Time 2). Moreover, Time 4 data were not used for the random intercept cross-lagged model because there were over 70% missing data in the Time 4 data. The large proportion of missing data affects the model specification even when imputation is conducted.

All random intercept cross-lagged models were performed using the diagonally weighted least square estimation. The fit indices with suggested cutoffs were comparative fit index (CFI) > 0.9, Tucker-Lewis index (TLI) > 0.9, root mean square error of approximation (RMSEA) < 0.08, and standardized root mean square residual (SRMR) < 0.08 (86–88). The descriptive statistics and Pearson correlations were performed using SPSS 24.0 (IBM Corp., Armonk, NY, USA), and the random intercept cross-lagged models were analyzed using LISREL 8.8 (Scientific Software International, Lincolnwood, IL, USA).

## Results

Table 1 presents the characteristics of all the participants. Given that not all the participants completed all the assessments, Table 1 also presents the characteristics of the participants who completed all assessments, those who missed one follow-up, those who missed two follow-ups, and those who missed three follow-ups. Attrition analyses further indicated that there were no significant differences in age, BMI, daily time spent on social media, and daily time spent gaming, BSMAS score, and IGDS-SF9 score (Wilks' λ = 1.00, F [4,582] = 1.26; *p* = 0.29). No significant difference was found in current cigarette smoking status [χ^2^ (1) = 2.58; *p* = 0.11]. However, more females were found among those who completed all the assessments (52.7%) than those who dropped out [74.0%; χ^2^ (1) = 24.07; *p* < 0.01].

**Table 1:** Participant characteristics.

	**Entire sample** ** (*N* = 601–645)**	**Sample that completed all three follow-ups** **(*n* = 162–178)**	**Sample that completed two follow-ups** **(*n* = 88–94)**	**Sample that completed one follow-up** **(*n* = 82-86)**	**Sample that completed no follow-ups** **(*n* = 269-287)**
Age; mean (SD)	20.95 (5.63)	21.19 (3.94)	20.96 (5.77)	21.82 (5.32)	20.51 (6.52)
Gender (Male); *n* (%)	266 (41.0%)	46 (26.0%)	28 (29.8%)	37 (43.5%)	155 (52.9%)
Time on social media (hours/day); mean (SD)	3.15 (2.74)	3.14 (2.65)	2.80 (2.28)	2.79 (1.87)	3.37 (3.10)
Time on gaming (hours/day); mean (SD)	1.17 (1.91)	1.09 (2.22)	0.95 (1.31)	1.43 (2.63)	1.22 (1.58)
Height (cm); mean (SD)	166.12 (8.62)	164.31 (8.16)	164.44 (9.04)	166.44 (8.78)	167.71 (8.42)
Weight (kg); mean (SD)	59.57 (12.88)	56.79 (10.31)	58.66 (18.02)	57.87 (10.48)	62.05 (12.52)
Body mass index (kg/m^2^); mean (SD)	21.43 (3.51)	20.92 (2.77)	21.45 (5.31)	20.82 (2.79)	21.92 (3.31)
BSMAS score	14.76 (4.26)	14.86 (4.23)	14.33 (4.07)	15.53 (3.97)	15.16 (4.33)
IGDS-SF9 score	16.92 (6.39)	17.69 (6.88)	17.18 (6.14)	16.36 (6.56)	16.48 (6.14)
HADS_ Anxiety score	6.37 (3.15)	5.82 (3.49)	6.12 (3.13)	6.15 (3.70)	5.70 (3.17)
HADS_ Depression score	5.04 (2.66)	4.51 (2.79)	5.00 (3.03)	4.93 (3.25)	4.18 (2.60)

*The sample sizes vary (e.g., N = 601**–**645) because some participants did not provide all their characteristics in the survey*.

After imputing the missing values, the average scores and the percentages above the clinical cut-off points on the BSMAS, IGDS-SF9, and HADS were calculated for each assessment time point (Table 2). More specifically, PSMU (assessed using the BSMAS) at baseline was found to be significantly higher than PSMU at other assessment time points (F = 4.03; *p* = 0.01). However, no significant differences were found between the four assessment time points in PG (assessed using the IGDS9-SF), anxiety and depression (both assessed using the HADS). The results in Table 3 further demonstrate the bivariate correlation coefficients between scores on the BSMAS, IGDS-SF9, and HADS for each assessment time point.

**Table 2:** Problematic social media use, problematic gaming, anxiety, and depression across time.

	**Mean (SD) % Above cut-off point**				**F (*p*-Value)**	***Post-hoc*** **comparison**
	**Time 1**	**Time 2**	**Time 3**	**Time 4**		
Problematic social media use	14.76 (4.26) 18.91%	14.04 (3.66) 11.16%	14.22 (3.90) 13.33%	14.16 (3.96) 13.64%	4.03 (0.01)	1>2, 1>3, 1>4
Problematic gaming	16.92 (6.39) 26.20%	17.01 (5.76) 24.95%	16.56 (5.40) 22.32%	17.01 (5.67) 25.89%	0.28 (0.77)	
Anxiety	6.37 (3.15) 18.37%	5.53 (3.08) 20.77%	6.02 (3.08) 25.73%	6.16 (3.08) 25.27%	0.47 (0.70)	
Depression	5.04 (2.66) 15.96%	4.13 (2.45) 8.37%	4.48 (2.73) 11.78%	4.34 (2.73) 11.47%	0.77 (0.51)	

*Problematic social media use assessed using the Bergen Social Media Addiction Scale; Problematic gaming assessed using the Internet Gaming Disorder Scale-Short Form; anxiety and depression assessed using the Hospital Anxiety and Depression Scale. Clinical cut-off point: Problematic social media use ≥19; Problematic gaming ≥21; Anxiety & depression ≥9; Time 1 = baseline; Time 2 = first follow-up; Time 3 = second follow-up; Time 4 = third follow-up*.

*Post-hoc comparison was done using the Bonferroni adjustment*.

**Table 3:** Correlation between problematic social media use, problematic gaming, and psychological distress (anxiety and depression).

	**r (** ***p*** **-Value)**							
	**PSMU_T1**	**PSMU_T2**	**PSMU_T3**	**PSMU_T4**	**PG_T1**	**PG_T2**	**PG_T3**	**PG_T4**
PSMU_T1	1.00							
PSMU_T2	0.76^**^	1.00						
PSMU_T3	0.75^**^	0.75^**^	1.00					
PSMU_T4	0.75^**^	0.74^**^	0.73^**^	1.00				
PG_T1	0.10^*^	0.12^**^	0.05	0.08^*^	1.00			
PG_T2	0.07	0.15^**^	0.14^**^	0.09^*^	0.79^**^	1.00		
PG_T3	0.06	0.16 ^**^	0.13^**^	0.09^*^	0.78^**^	0.79^**^	1.00	
PG_T4	0.17^**^	0.24^**^	0.17^**^	0.23^**^	0.78^**^	0.73^**^	0.73^**^	1.00
A_T1	0.15^**^	0.15^**^	0.23^**^	0.17^**^	0.26^**^	0.27^**^	0.24^**^	0.26^**^
A_T2	0.18^**^	0.23^**^	0.23^**^	0.24^**^	0.23^**^	0.29^**^	0.21^**^	0.36^**^
A_T3	0.30^**^	0.26^**^	0.37^**^	0.32^**^	0.17^**^	0.20^**^	0.19^**^	0.27^**^
A_T4	0.29^**^	0.21^**^	0.31^**^	0.34^**^	0.16^**^	0.21^**^	0.16^**^	0.34^**^
D_T1	0.18^**^	0.17^**^	0.19^**^	0.14^**^	0.26^**^	0.27^**^	0.32^**^	0.36^**^
D_T2	0.15^**^	0.17^**^	0.18^**^	0.17^**^	0.14^**^	0.26^**^	0.19^**^	0.34^**^
D_T3	0.24^**^	0.19^**^	0.27^**^	0.22^**^	0.25^**^	0.31^**^	0.32^**^	0.39^**^
D_T4	0.29^**^	0.23^**^	0.26^**^	0.30^**^	0.20^**^	0.24^**^	0.22^**^	0.42^**^

* *Correlation is significant at the 0.05 level (2-tailed);*

** *Correlation is significant at the 0.01 level (2-tailed)*.

*PSMU, problematic social media use assessed using the Bergen Social Media Addiction Scale; PG, problematic gaming assessed using the Internet Gaming Disorder Scale-Short Form; A, anxiety and D, depression, both assessed using the Hospital Anxiety and Depression Scale; T1, Time 1 (baseline); T2, Time 2 (first follow-up); T3, Time 3 (second follow-up); T4, Time 4 (third follow-up)*.

**Figure 1:**
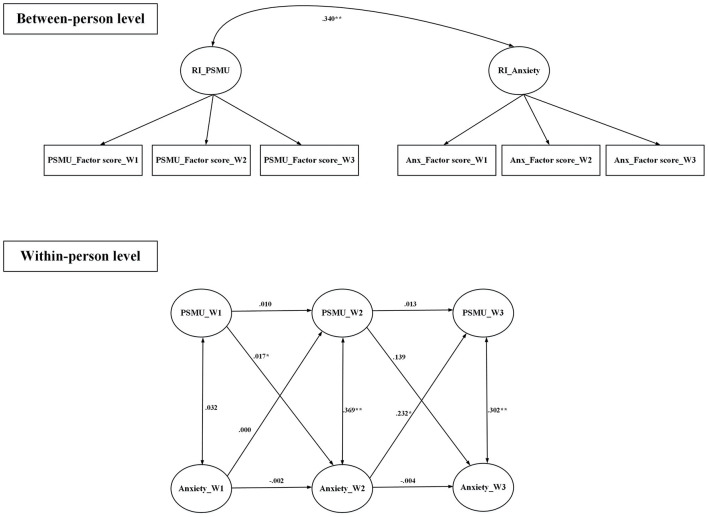
Cross-lagged model of PSMU and anxiety using lagged bidirectional model. Covariates include gender and age. Anxiety was assessed using the Hospital Anxiety and Depression Scale; PSMU, problematic social media use was assessed using the Bergen Social Media Addiction Scale. RI, random intercept; W1, wave 1; W2, wave 2; W3, wave 3. All the estimates are standardized coefficients. *p < 0.05; **p < 0.01.

**Figure 2:**
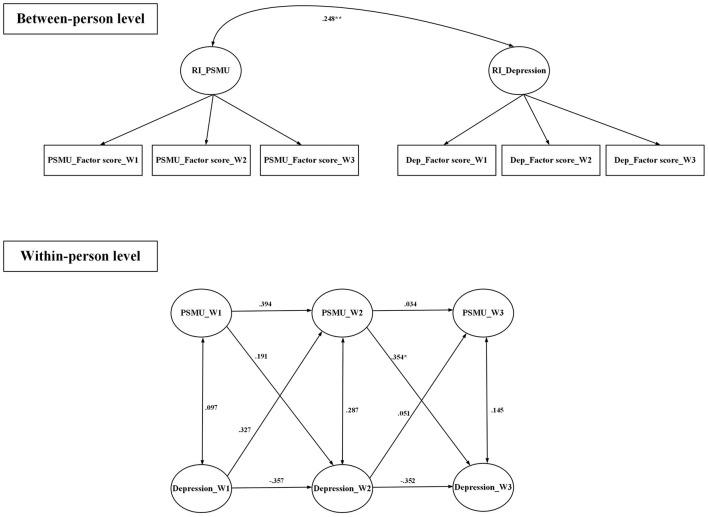
Cross-lagged model of PSMU and depression using lagged bidirectional model. Covariates include gender and age. Depression was assessed using the Hospital Anxiety and Depression Scale; PSMU, problematic social media use was assessed using the Bergen Social Media Addiction Scale. RI, random intercept; W1, wave 1; W2, wave 2; W3, wave 3. All the estimates are standardized coefficients. *p < 0.05; **p < 0.01.

**Figure 3:**
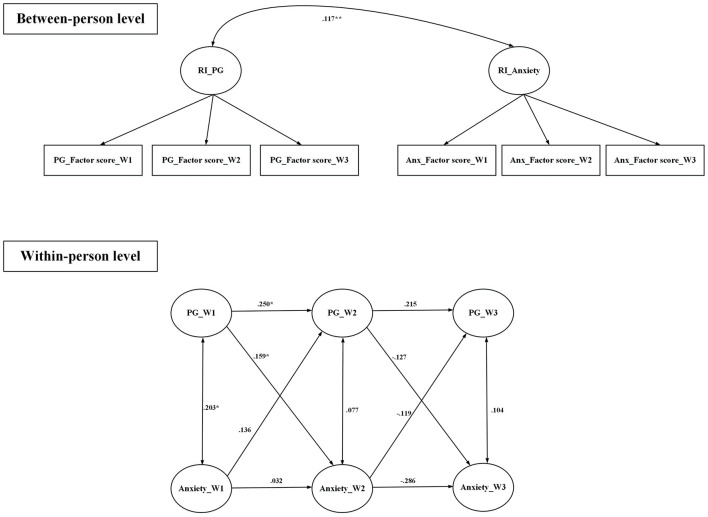
Cross-lagged model of PG and anxiety using lagged bidirectional model. Covariates include gender and age. Anxiety was assessed using the Hospital Anxiety and Depression Scale; PG, problematic gaming was assessed using the Internet Gaming Disorder Scale-Short Form. RI, random intercept; W1, wave 1; W2, wave 2; W3, wave 3. All the estimates are standardized coefficients. *p < 0.05; **p < 0.01.

**Figure 4:**
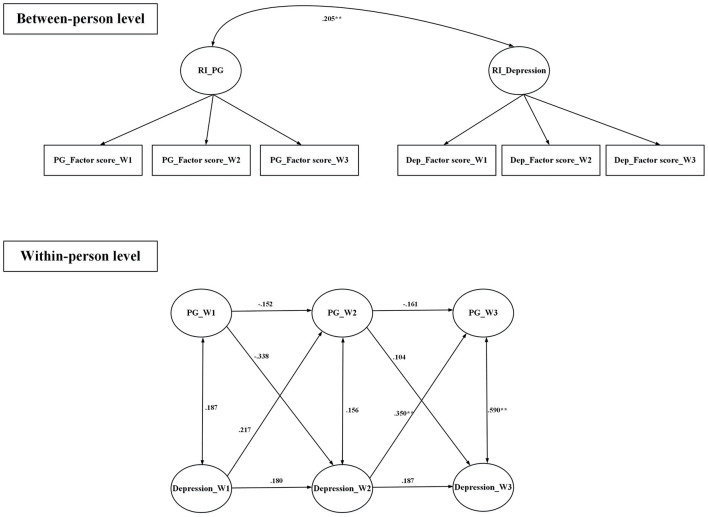
Cross-lagged model of PG and depression using lagged bidirectional model. Covariates include gender and age. Depression was assessed using the Hospital Anxiety and Depression Scale; PG, problematic gaming was assessed using the Internet Gaming Disorder Scale-Short Form. RI, random intercept; W1, wave 1; W2, wave 2; W3, wave 3. All the estimates are standardized coefficients. *p < 0.05; **p < 0.01.

All fit indices were satisfactory for the random intercept cross-lagged models that examined the relationships between (i) anxiety and PSMU, (ii) anxiety and PG, (iii) depression and PSMU, and (iv) depression and PG (Table 4). Figure 1 demonstrates that PSMU significantly impacted on anxiety, and then anxiety significantly impacted on PSMU; Figure 2 demonstrates that PSMU significantly impacted on depression, but depression did not impact on PSMU. Figure 3 demonstrates that PG significantly impacted on anxiety, but anxiety did not impact PG; Figure 4 demonstrates that PG did not significantly impact depression, but depression did impact PG.

**Table 4:** Fit indices of the random intercept cross-lagged models.

	**Anxiety and PSMU**	**Depression and PSMU**	**Anxiety and PG**	**Depression and PG**
χ^2^ (df)	0.017 (4)	11.915 (4)	12.930 (4)	6.327 (4)
CFI	0.999	0.998	0.999	0.999
TLI	0.999	0.993	0.978	0.994
RMSEA	0.000	0.031	0.021	0.030
SRMR	0.002	0.030	0.011	0.012

*PSMU, problematic social media use assessed using the Bergen Social Media Addiction Scale; PG, problematic gaming assessed using the Internet Gaming Disorder Scale-Short Form; anxiety and depression were assessed using the Hospital Anxiety and Depression Scale*.

*CFI, comparative fit index; TLI, Tucker-Lewis index; RMSEA, root mean square error of approximation; SRMR, standardized root mean square residual*.

*Anxiety and PSMU model shown in Figure 1; Depression and PSMU model shown in Figure 2; Anxiety and PG model shown in Figure 3; Depression and PG model shown in Figure 4*.

## Discussion

To the best of the present authors' knowledge, the present study is the first to use random intercept cross-lagged models to examine the reciprocal relationship between PSMU, PG, and two types of psychological distress (depression and anxiety) among university students across three time points. Findings showed that a high level of anxiety might lead to a high level of PSMU, and, reciprocally, a high level of PSMU might lead to a high level of anxiety. Furthermore, a high level of PSMU might lead to a high level of depression but not in the opposite direction. The present study's findings also showed that a high level of PG might lead to a high level of anxiety, but not in the opposite direction. Moreover, a high level of depression might lead to a high level of PG, but not in the opposite direction. Additionally, analysis of the longitudinal data indicated there were no differences across the different waves of the study in terms of gaming by the participants.

The findings of Li et al.'s (51) study indicated that the relationship between PSMU and depression was bi-directional. The present study's findings also suggest that a high level of PSMU leads to an increased depression level. PSMU can inhibit relationships with family members and friends in the real world and can lead to social isolation, resulting in depression (40). Increased time spent on social media use can also impact negatively on education (e.g., lower grades) and further leads to a high level of depression (38). However, in the present study, a high depression level did not increase the level of PSMU. This finding contradicts that of Li et al. (51), who reported that depression could lead to PMSU. The difference in study samples might explain the inconsistent findings between Li et al.'s and the present study. More specifically, the participants were high school students in Li et al.'s study (51), whereas the participants in the present study were university students. It is possible that university students have more diverse social networks and strategies to cope with depressive feelings than high school students. Moreover, to use social media, university students might use other ways for coping when they feel depressed. Therefore, while Li et al.'s study findings showed that the depression level was associated with increased the PSMU level in high school students, the present study did not find such an association. Future research on the moderating effects of age groups in the causal relationship between PSMU and depression levels is needed.

The results of the present study also showed that a high anxiety level could cause a high level of PSMU and, reciprocally, a high level of PSMU could cause a high level of anxiety. As the present authors are not aware of any other longitudinal study testing the effect of anxiety upon social media use, the present study enhances knowledge on the causal relationship between these two variables. Possibly, those with high anxiety levels tend to be afraid of missing out on online information, opportunities for online social interactions, and online rewarding experiences (89). In turn, the fear leads to a high level of PSMU.

Although there are similarities between the present study and the previous study findings (56), some of findings are different. In the previous and the present study findings, a high level of PG was associated with an increased level of anxiety; and a high level of depression was related to an increased level of PG. However, while the previous study finding showed that the level of anxiety was related to an increased level of pathological gaming, the present study findings indicated that the level of anxiety did not impact the level of PG. Potentially, university students, the sample in the present study, were more mature and more capable of coping with anxiety compared with adolescents (i.e., the samples used in previous studies), because they may have more social resources and autonomy. Consequently, to deal with anxiety, university students are more likely to choose other means, rather than online gaming, to cope with their anxiety.

Utilizing a longitudinal study design, the findings of the present study indicated that both PSMU and PG could lead to high levels of anxiety. These findings infer that the awareness of high levels of problematic internet use might create high levels of anxiety. However, the reciprocal relationship only occurs between PSMU and anxiety, in which high levels of anxiety lead to high levels of PSMU, but not PG. Given that the characteristics of social media use and online gaming differ, the findings are not surprising. Because social media use typically involves more social interaction and emotional effort than online gaming, potentially, the anxiety arising from social media use is associated with negative social experiences on social media. Consequently, users spend more time and effort on social media to cope with anxiety pertaining to social media use. As a result, high levels of anxiety would, in turn, increase the level of PSMU. However, the anxiety arising from online gaming might not involve social relationship issues and, therefore, participants can choose other strategies to deal with online gaming-related anxiety. As a result, there was no reciprocal relationship between PG and anxiety in the present study. As indicated in the study results, while participants spent ~3 h on social media daily, they spent ~1 hour online gaming per day. Compared to online gaming, more time spent on social media use also reflected the reciprocal process.

As indicated in Table 2, only a small proportion of study participants reached clinical levels for psychological distress (i.e., depression, anxiety) and PIU (i.e., PSMU, PG) and the average times spent on social media and online gaming were relatively reasonable. Therefore, it requires further investigation to verify whether the findings of the present may reflect the experiences for heavy internet users or those with clinical depression and anxiety. However, since there are differences on each variable, the associations found in the present study have some practice implications. Given that high levels of PSMU and PG could cause high levels of psychological distress among young adults, colleges or universities should introduce prevention programs on digital health and mental wellbeing to minimize serious mental health problems occurring among their students. Using psychological interventions, such as cognitive-behavioral therapy to break the reciprocal relationship between PSMU and anxiety would be imperative in the interventions for young adults experiencing PSMU. In addition, because social media use is now commonplace among young adults, when working with young adults with depression and anxiety, clinicians should be aware of the potential effects of problematic social media use. Screening the level of PSMU and, if the level of PSMU is high, addressing issues related to it should be considered as part of the intervention plan for young adults with depression. Moreover, the auto-regressive paths in the analyzed models were not significant. This indicated that previous anxiety and depression among university students were not associated with their later anxiety and depression. Therefore, healthcare providers should not heavily rely on students' prior psychological distress to estimate their future psychological health problems.

There are some limitations in the present study. First, the majority of the participants in the present study were Hong Kong or Taiwanese university students and they were recruited through a convenience sampling method. Therefore, the representativeness of the present sample was restricted. Second, the attrition rate in the present study was arguably large (nearly three-quarters of the participants at baseline did not complete all three follow-up assessments). Therefore, the present study's findings may somewhat be biased. Nevertheless, multiple imputations (based on MCMC method) were used to take care of the missing cases. Third, instead of objective measures, only self-report questions were used to evaluate the present study's variables, including PIU, psychological distress, weight, and height. Therefore, such measures are subject to recall bias, social desirability, and single-rater bias. Finally, only PG and PSMU were assessed in the present study. Future studies may want to investigate the relationship between other specific forms of problematic use in internet-related activities (e.g., problematic online shopping) and psychological distress, using larger and more nationally representative samples.

## Conclusion

The present study found a reciprocal relationship between PSMU and anxiety. In addition, unidirectional relationships between PSMU, PG, and psychological distress were identified. More specifically, high PSMU levels led to high depression levels; high PG levels led to high anxiety levels; and high depression levels led to high PG levels. Moreover, the longitudinal design found no differences in the waves in terms of gaming by the participants. With the aforementioned findings, the present study provides mental health professionals with useful information when designing and implementing program and interventions to improve psychological health and wellbeing among young adults.

## Data Availability Statement

The raw data supporting the conclusions of this article will be made available by the authors, without undue reservation.

## Ethics Statement

The research proposal was approved by the Ethics Committee of the Hong Kong Polytechnic University's Ethics Committee (Ref. No. HSEARS20171212001). The patients/participants provided their written informed consent to participate in this study.

## Author Contributions

C-WC, R-YH, I-HC, and C-YL created and organized the study, wrote the first draft, analyzed, and interpreted the data. C-YL, CS, Y-CL, and M-CT collected the data. I-HC analyzed the data. AP provided the directions of data analysis. MG supervised the entire study and was responsible for all final editing. AP, R-YH, C-YL, CS, Y-CL, M-CT, I-HC, and MG critically reviewed the manuscript and provided constructive comments. All authors contributed to the article and approved the submitted version.

## Funding

This research was supported in part by (received funding from) the startup fund in the Department of Rehabilitation Sciences, The Hong Kong Polytechnic University, Hong Kong; the Ministry of Science and Technology, Taiwan (MOST 110-2410-H-006-115); 2020 Jiangxi Social Science Foundation Project: Online Synchronous Psychological Groups Integration Research (20JY07), and 2020 General Research Project of Humanities and Social Science for Colleges and Universities in Jiangxi Province: Application and Development of Network Psychological Groups from the Perspective of Ecosystem, Youth Project (XL20205).

## Conflict of Interest

The authors declare that the research was conducted in the absence of any commercial or financial relationships that could be construed as a potential conflict of interest.

## Publisher's Note

All claims expressed in this article are solely those of the authors and do not necessarily represent those of their affiliated organizations, or those of the publisher, the editors and the reviewers. Any product that may be evaluated in this article, or claim that may be made by its manufacturer, is not guaranteed or endorsed by the publisher.
